# Supplementary material for: Mechanical Disturbance of Osteoclasts Induces ATP Release That Leads to Protein Synthesis in Skeletal Muscle through an Akt-mTOR Signaling Pathway
Source: Int J Mol Sci. 2022 Aug 21;23(16):9444. doi: 10.3390/ijms23169444 (PMC9408906; doi:10.3390/ijms23169444)
Supplement: Supplementary file 1 [file ijms-23-09444-s001.zip › ijms-1858400-supplementary.pdf]

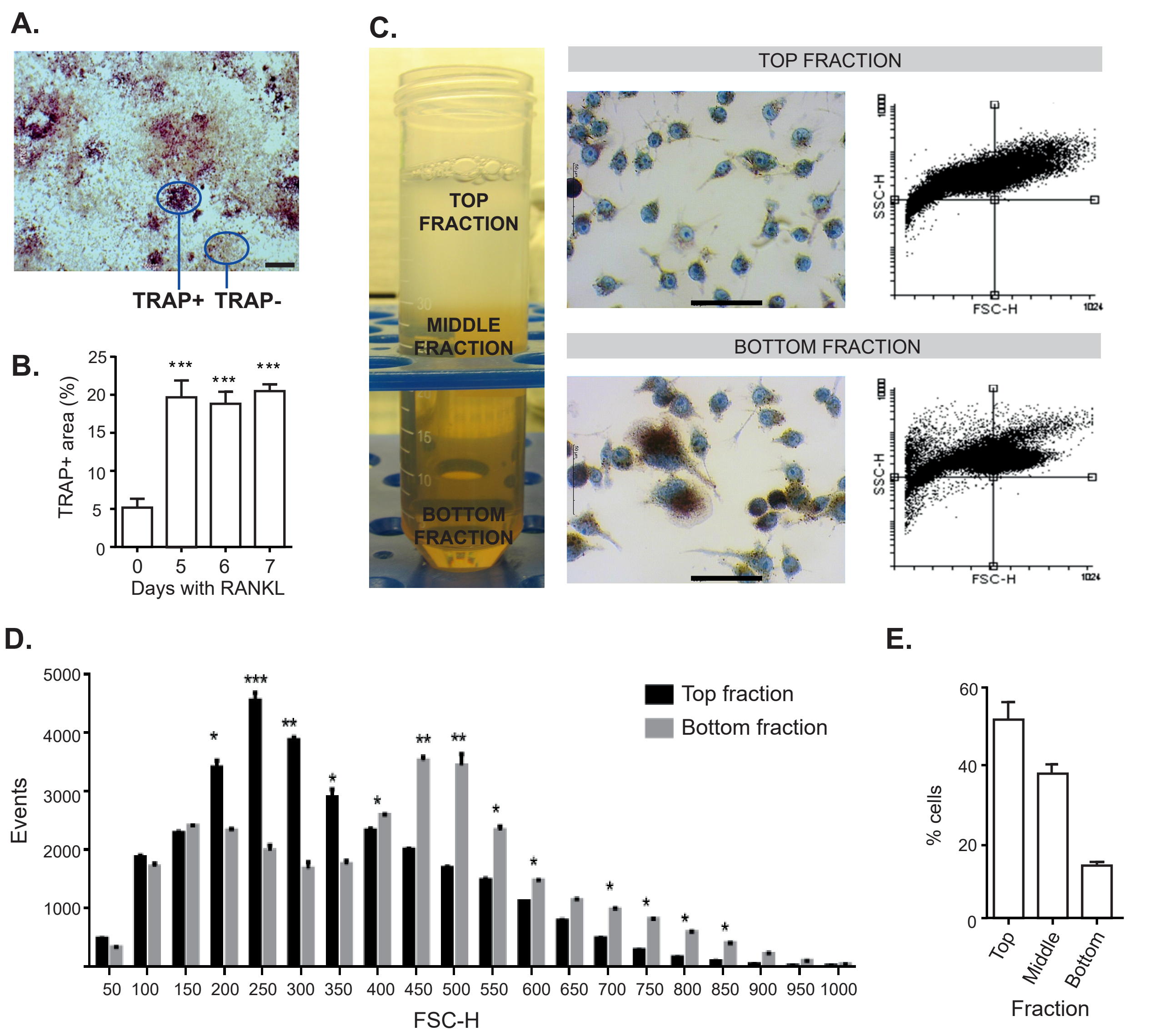

Supplementary Figure S1, Morales-Jiménez et al 2022

Supplementary Figure S1. Characterization of an osteoclast-enriched fraction by serum gradient.
A. TRAP staining in cell culture of RAW 264.7 cells incubated with 35 ng/ml RANKL for 5d (4X objective, scale bar 200 µm, representative image). **B.** Quantification of the percentage of the area with TRAP+ staining (relative to the total cell area) after 5, 6, and 7 days of RANKL. n=6, *** p<0.001, Kruskal Wallis test followed by Bonferroni post hoc, comparing each time against the 0. **C.** Considering that no more than 20% of the total cell area was differentiated into osteoclasts with RANKL, a subsequent step of purification with a serum gradient was used, as depicted in the left panel. Middle panels show TRAP staining and morphology of cells obtained from top or bottom fractions (scale bar 50 µm). An enrichment in TRAP+/large/multinucleated-cells is observed in the bottom fraction. Right panels are representative tracings of the distribution of cells from the top and bottom fractions after fluorescence-activated cell sorting. In each condition, 30.000 cells were sorted according to the size (FSC-H) and internal complexity/granularity (SSC-H). **D.** Histogram of cell number vs. FSC-H frontal dispersion derived from flow cytometry for the top fraction (black bars) and the bottom fraction (gray bars). n = 3. Average ± SEM. *: p <0.05, **: p <0.01, ***: p <0.001, Mann-Whitney test comparing top and bottom fraction for each FSC-H value. **E.** Percentage of cells in each collected-fraction of the serum gradient, relative to the total cell number.

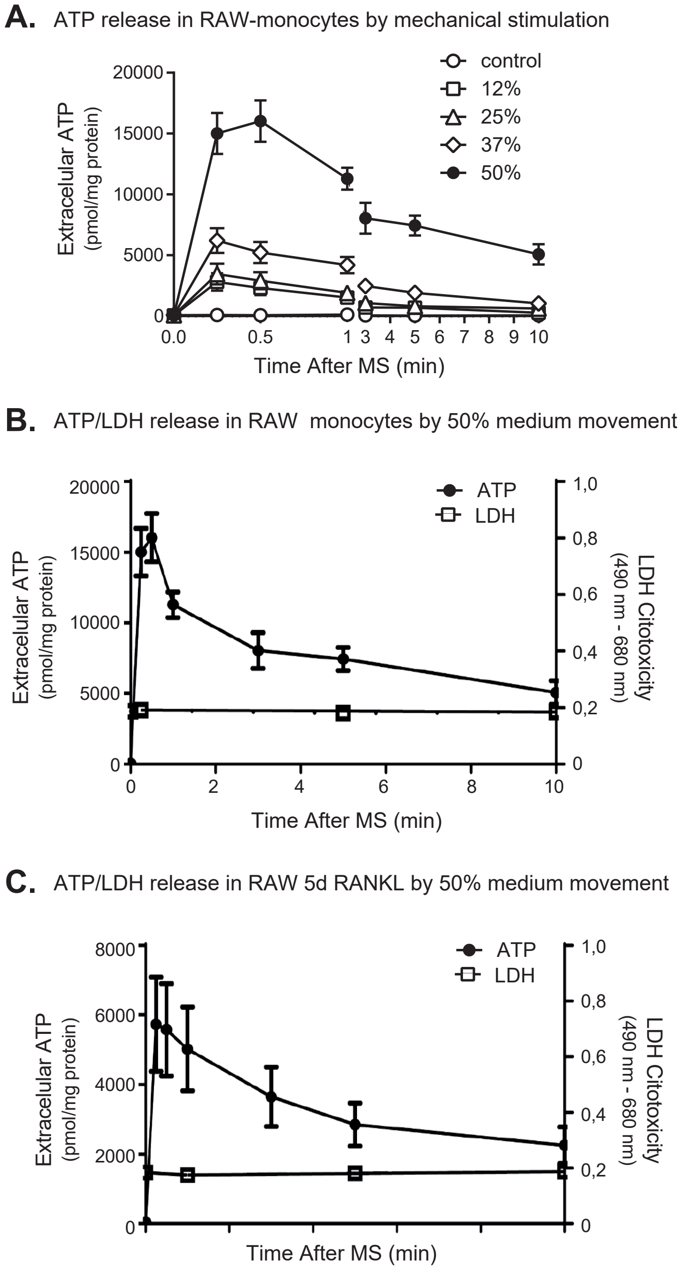

Supplementary Figure S2, Morales-Jiménez et al 2022

Supplementary Figure S2. Controls of ATP-release experiments

A. Mechanical stimulation evoked ATP release in RAW 264.7 cells at the monocyte state. Cells were mechanically stimulated (MS) by pipetting different volumes of the extracellular medium (12, 25, 37, 50% of the total amount; one pipetting per second, ten times). The control condition corresponds to unperturbed cells. At different times post-stimulation, extracellular ATP was measured at the cell media using a luciferin-luciferase kit. **B-C Mechanical stimulation of RAW 264.7 cells did not lead to cell lysis, either in the monocyte state (B) or after differentiation with RANKL for 5d (C).** To address a putative cell lysis effect, the maximal mechanical stimulation (MS) was addressed (movement of 50% of the extracellular medium). LDH activity was measured in the extracellular aliquots at different times after the stimuli, in parallel to the ATP measurements. No increase in LDH activity was observed at any time. n = 6. Mean ± SEM.

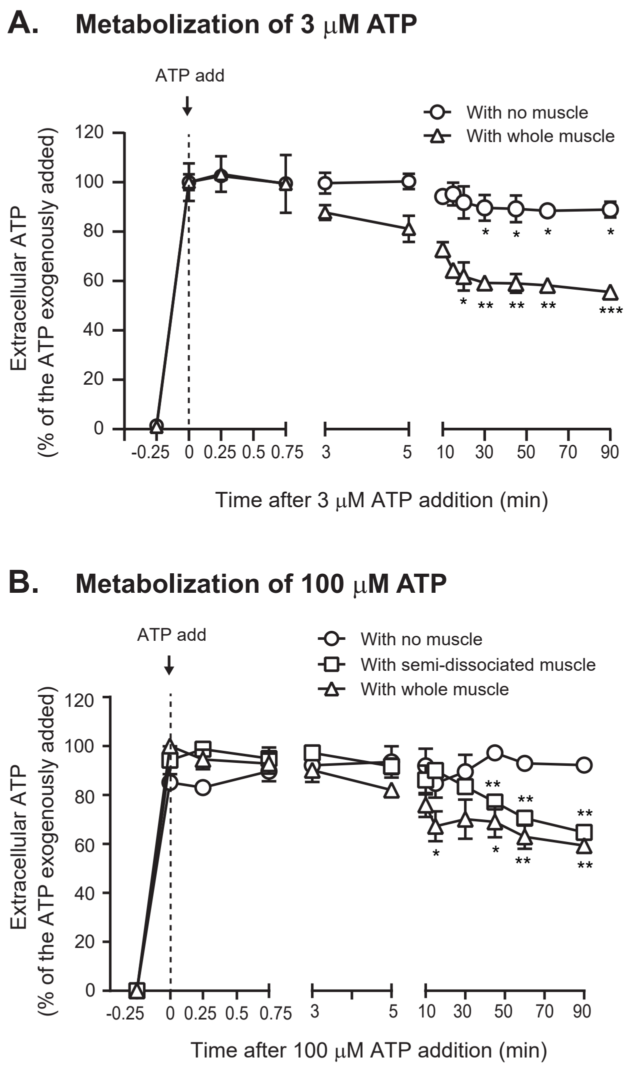

Supplementary Figure S3, Morales-Jiménez et al 2022

Supplementary Figure S3. Kinetics of exogenous-ATP metabolization by FDB muscles.

The concentration of extracellular ATP was quantified at different times after addition of 3 μM ATP **(A)** or 100 μM ATP **(B)** to 35mm plates without muscle (control), with whole FDB muscle, or with previously semi-dissociated FDB muscle (collagenase IV, 30 min). n = 3. Mean ± SEM. *: $p < 0.05$; **: $p < 0.01$; Kruskal Wallis test followed by Bonferroni post hoc, comparing each time against the 0.

Supplemental Table S1. qRT-PCR primers

Gene	Concentration	Forward	Reverse
GAPDH [1]	400 nM	CAACTTTGGCATTGTGGAAG	CTGCTTCACCACCTTCTTG
TRAP [2]	600 nM	CGACCATTGTTAGCCACATACG	TCGTCCTGAAGATACTGCAGGTT
Cathepsin K [2]	600 nM	ATATGTGGGCCAGGATGAAAGTT	TCGTTCCCCACAGGAATCTCT
Carbonic Anhydrase [3]	400 nM	CTCTGCTGGAATGTGTGACCT	CTGAGCTGGACGCC AGTTGTC
Integrin β3	600 nM	GGAAGAACTGTCACTGTCCT	ACTAAAGCTCACCGTGTCTC.
Lysosomal ATPase [3]	600 nM	ACGGTGATGTCACAGCAGACGT	CCTCTGGATAGAGCCTGCCGCA.
Metalloproteinase-9 [3]	600 nM	GCTGACTACGATAAGGACGGCA	GCGGCCCTCAAAGATGAACGG
$P2X_1$ [4]	200	TGTACGGGGAGAAGAACCTG	TCCCAAACACCTTGAAGAGG
$P2X_2$ [4]	600	CGTCTTCATCGTGCAGAAAA	CACTTTGTGTTCCGACATGG
$P2X_3$ [4]	400	TACCAAGTCGGTGGTTGTGA	CCACCCCACAAAGTAGGAGA
$P2X_4$ [4]	600	GCACCCTCCACCATCTCTAA	AAACCTCTTGCCAGAAGCAA
$P2X_5$ [4]	400	ACCAACCTGATCGTGACTCC	CAGTCGGTGTCCTCTGAACA
$P2X_6$ [4]	400	GTGGATAGAGAGGCCGGTTT	GTAACAGGGTTAGCGGGTGA
$P2X_7$ [4]	600	GGCACTGGAGGAAAATTTGA	TGAGCAAGTCAATGCACACA
$P2Y_1$ [4]	400	AGGAAAGCTTCCAGGAGGAG	CGTGTCTCCATTCTGCTTGA
$P2Y_2$ [4]	600	GTCAGCAGTGACGACTCAAGAC	TCAGAGGATATCAGCCCCTTTA
$P2Y_4$ [4]	600	AGGAAGCAGCAGAACACCAT	CAAGGAGTCTGCACTGGTCA
$P2Y_6$ [4]	600	TTCCATCTTGCATGAGACAGAC	GCTTGAAATCCTCACGGTAGAC
$P2Y_{12}$ [4]	600	CCTGTGCGTCAGAGACTACAAG	GGATTTACTGCGGATCTGAAAG
$P2Y_{13}$ [4]	600	GGCCACTAGATGTCACCTTTTC	GATGGTGGGGTGGTAACTAGAA
$P2Y_{14}$ [4]	600	GGAATTCTCTCTTCCGAATCCT	TGTTCATCTTCTCACCTCTGGA

1. Bustamante M, Fernandez-Verdejo R, Jaimovich E, Buvinic S. Electrical stimulation induces IL-6 in skeletal muscle through extracellular ATP by activating Ca(2+) signals and an IL-6 autocrine loop. Am J Physiol Endocrinol Metab. Apr 15 2014;306(8):E869-82.
2. Swanson C, Lorentzon M, Conaway HH, Lerner UH. Glucocorticoid regulation of osteoclast differentiation and expression of receptor activator of nuclear factor-kappaB (NF-kappaB) ligand, osteoprotegerin, and receptor activator of NF-kappaB in mouse calvarial bones. Endocrinology. Jul 2006;147(7):3613-22.
3. Arriero Mdel M, Ramis JM, Perello J, Monjo M. Inositol hexakisphosphate inhibits osteoclastogenesis on RAW 264.7 cells and human primary osteoclasts. PLoS One. 2012;7(8):e43187.
4. Orriss IR, Wang N, Burnstock G, Arnett TR, Gartland A, Robaye B, et al. The P2Y(6) receptor stimulates bone resorption by osteoclasts. Endocrinology. Oct 2011;152(10):3706-16.

Supplemental Table S2.

Detection of mRNA for different P2Y/P2X receptor subtypes in RAW 264.7 cells, in monocyte or osteoclast state.

Receptor subtype	RAW 264.7 monocyte	RAW 264.7 osteoclast
$P2X_1$	-	-
$P2X_2$	-	-
$P2X_3$	-	+
$P2X_4$	+	+
$P2X_5$	-	-
$P2X_6$	-	-
$P2X_7$	+	+
$P2Y_1$	-	-
$P2Y_2$	+	+
$P2Y_4$	-	-
$P2Y_6$	+	+
$P2Y_{12}$	+	+
$P2Y_{13}$	-	-
$P2Y_{14}$	-	-

RAW 264.7 cells were maintained for 5d with regular medium (RAW 264.7 monocyte) or supplemented with 35 ng/ml RANKL (RAW 264.7 osteoclast). Total RNA was isolated, and cDNA was obtained by reverse transcription. qPCR was developed using selective primers for P2Y/P2X receptor subtypes (see Material and Methods Supplemental Tables), with GAPDH as the housekeeping gene. Detected (+) or non-detected (-) expression is reported. n= 3-5

Supplemental Table S3. List of antibodies used for western blotting (wb) or immunofluorescence (IF)

Epitope/Antigen or Product name	Source and Catalog # or RRID	Host species	Application (WB, IP, F, IHC, ICC, CHIP, N)	Dilution	Application-specific details (e.g., antigen retrieval, blocking, incubation)
GAPDH	Cat# sc-32233, RRID:AB_627679	Mouse	WB	1/10000	5% BSA, 2 hours, 4°C
Akt	Cat# 2920, RRID:AB_1147620	Mouse	WB	1/1000	5% BSA, overnight, 4°C
Phospho-Akt (Ser473)	Cat# 11962, RRID:AB_2797780	Rabbit	WB	1/2000	5% BSA, overnight, 4°C
mTOR (7C10)	Cat# 5043, RRID:AB_10693441	Rabbit	WB	1/1000	5% BSA, overnight, 4°C
Phospho-mTOR (Ser2448)	Cat# 2971, RRID:AB_330970	Rabbit	WB	1/1000	5% BSA, overnight, 4°C
Phospho-p70 S6 Kinase (Thr389)	Cat# 9025, RRID:AB_2734746	Rabbit	WB	1/1000	5% BSA, overnight, 4°C
S6 Ribosomal Protein (5G10)	Cat# 2217, RRID:AB_331355	Rabbit	WB	1/1000	5% BSA, overnight, 4°C
Phospho-S6 Ribosomal Protein (Ser235/236)	Cat# 2211, RRID:AB_331679	Mouse	WB	1/2000	5% BSA, overnight, 4°C
Phospho-4E-BP1 (Thr37/46)	Cat# 9459, RRID:AB_330985	Rabbit	WB	1/1000	5% BSA, overnight, 4°C
Puromycin Antibody, clone 12D10	Cat# MABE343, RRID:AB_2566826	Mouse	WB	1/5000	5% BSA, overnight, 4°C
$P2X_7$ receptor	sc-134224	rat	IF	1/50	5% BSA, overnight, 4°C
Alexa Fluor 555 Dye	AB_2535855	Goat	IF	1/500	5% BSA, 1 h, 37°C

Supplemental Table S4. Cell Lines and Animals

**Cell Line**	***Source and catalog# or RRID***	**Proving laboratory**	**Species, cell Type**
RAW 264.7	*ATCC®, TIB-71™*	Dr. Mario Galindo, Faculty of Medicine, Universidad de Chile	Mouse macrophage
**Mouse model**	**Source and catalog#**	**strain**	**Providing Laboratory**
Wild type mice	Cat# JAX:000651, RRID:IMSR_JAX:000651	BALB/c Mus musculus	NA

Supplemental Table S5. Biological modulators used

Modulator (protein, small molecule)	Source, catalog # or RRID	Solvent/Vehicle	Concentration(s)
Adenosine 5′-triphosphate disodium salt hydrate	CAS # 34369-07-8	Milli Q Water	0.1-100 μM
CellTiter-Glo® 2.0 Assay	CAS # G9241	NA	NA
Recombinant Human TRANCE/RANK L/TNFSF11 Protein	CAS # 390-TN-010	Sterile PBS containing at least 0.1% human or bovine serum albumin.	35 ng /mL
Puromycin Dihydrochloride	CAS # 58-58-2	DMEM	1 μM
Suramin sodium salt	CAS # 129-46-4	Milli Q Water	100 μM
Akt Inhibitor VIII	CAS # 124036	DMSO	1 μM
Rapamycin	CAS # R8781	DMSO	100 nM
LY294002	CAS # PHZ1144	DMSO	50 μM
